# Healthy lifestyles reduce suPAR and mortality in a Danish general population study

**DOI:** 10.1186/s12979-018-0141-8

**Published:** 2019-01-22

**Authors:** Thomas Huneck Haupt, Line Jee Hartmann Rasmussen, Thomas Kallemose, Steen Ladelund, Ove Andersen, Charlotta Pisinger, Jesper Eugen-Olsen

**Affiliations:** 1 Clinical Research Centre, Amager and Hvidovre Hospital, Hvidovre, Denmark; 2 Faculty of Health and Medical Sciences, University of Copenhagen, Copenhagen, Denmark; 3 Department of Orthopedic Surgery, Amager and Hvidovre Hospital, Hvidovre, Denmark; 4 Centre for Clinical Research and Prevention, Bispebjerg and Frederiksberg Hospital, Frederiksberg, Denmark; 5 Department of Public Health, Faculty of Health and Medical Sciences, University of Copenhagen, Copenhagen, Denmark

**Keywords:** Chronic inflammation, Prognosis, Biological ageing, Biomarker, Risk, Lifestyle change, Impact, Diet, Smoking, Exercise

## Abstract

**Background:**

The plasma level of the inflammatory biomarker soluble urokinase plasminogen activator receptor (suPAR) is a strong predictor of disease development and premature mortality in the general population. Unhealthy lifestyle habits such as smoking or unhealthy eating is known to elevate the suPAR level. We aimed to investigate whether change in lifestyle habits impact on the suPAR level, and whether the resultant levels are associated with mortality.

**Results:**

Paired suPAR measurements from baseline- and the 5-year visit of the population-based Inter99 study were compared with the habits of diet, smoking, alcohol consumption, and physical activity. Paired suPAR measurements for 3225 individuals were analyzed by linear regression, adjusted for demographics and lifestyle habits. Compared to individuals with a healthy lifestyle, an unhealthy diet, low physical activity, and daily smoking were associated with a 5.9, 12.8, and 17.6% higher 5-year suPAR, respectively. During 6.1 years of follow-up after the 5-year visit, 1.6% of those with a low suPAR (mean 2.93 ng/ml) died compared with 3.8% of individuals with a high suPAR (mean 4.73 ng/ml), *P* <  0.001. In Cox regression analysis, adjusted for demographics and lifestyle, the hazard ratio for mortality per 5-year suPAR doubling was 2.03 (95% CI: 1.22–3.37).

**Conclusion:**

Lifestyle has a considerable impact on suPAR levels; the combination of unhealthy habits was associated with 44% higher 5-year suPAR values and the 5-year suPAR was a strong predictor of mortality. We propose suPAR as a candidate biomarker for lifestyle changes as well as the subsequent risk of mortality.

## Background

Lifestyle intervention is an integral part of the management of common chronic diseases, including cardiovascular disease (CVD), type 2 diabetes, and chronic obstructive pulmonary disease [1–3]. Achieving and maintaining a healthy lifestyle is challenging and the benefit is often merely an abstract reduction in the risk of future disease, which may be insufficient motivation for sustaining a healthy lifestyle. However, individuals’ knowledge about their risk improves the adherence to lifestyle changes [4–7]. In the 1990s, inflammation was recognized as a major risk factor for the development of classical lifestyle diseases [8–11]. The gold standard for measuring inflammation in healthy persons is C-reactive protein (CRP) measured using a highly sensitive assay. However, while CRP is a marker for CVD risk, an indicator for mortality risk may be more appropriate when assessing overall health. Also, CRP may lack intra-individual stability [12–15]. Better biomarkers for risk stratification may lead to more appropriate lifestyle interventions and, thus, prevention of disease.

The urokinase plasminogen activator receptor (uPAR) is expressed on activated immune cells and involved in cell adhesion and migration, proliferation and invasion through degradation of the extracellular matrix [16]. Under inflammatory conditions, uPAR is released from the cell surface generating a soluble form of the receptor (suPAR) with intrinsic chemotactic properties [17]. suPAR is a stable biomarker, both in vivo and in vitro [18, 19], and is positively correlated with labile pro-inflammatory cytokines such as IL-1β and TNF-α as well as with C-reactive protein [20]. Elevated levels are associated with increased risk of CVD, type 2 diabetes, chronic kidney disease, cancer, and premature death in the general population [21–24]. We have previously shown that suPAR levels are associated with lifestyle [25], but it is currently unknown if lifestyle changes are also reflected in subsequent suPAR measurements and if such changes affect an individual’s mortality risk. If so, suPAR could be used for guiding lifestyle changes, similar to pharmacological interventions against hypercholesterolemia or hypertension. To address these questions, we investigated if lifestyle changes during a 5-year period affected suPAR levels in a randomized, population-based study. Furthermore, we aimed to investigate if the resultant suPAR levels were associated with mortality.

## Results

### Population characteristics

**Fig. 1:**
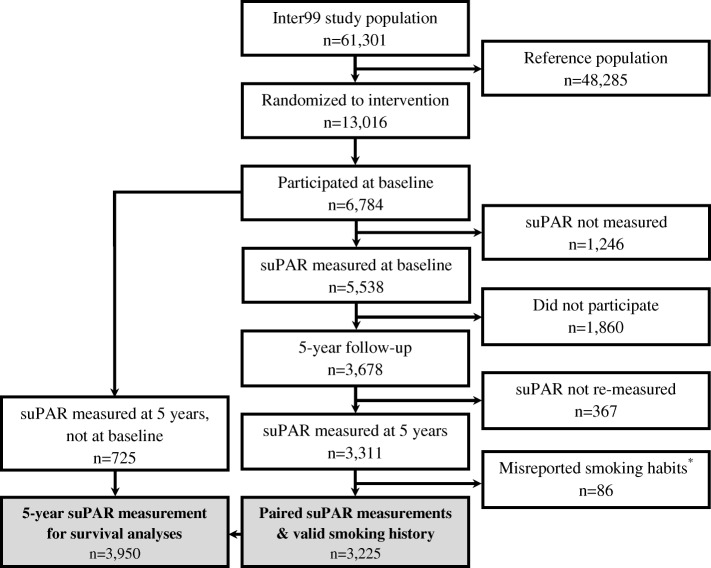
Flow of study participants. The “misreported smoking” group includes the one participant who was a never smoker at baseline and daily smoker at the 5-year visit.

The study included 3225 individuals with suPAR measured at baseline and at 5-year follow-up (see Fig. 1 and Methods for details on study flow). From baseline to the 5-year visit, 11% of participants adopted a healthy diet, about 8% quit smoking, and 5% fewer had a low level of physical activity (Table 1). Alcohol consumption was stable, with about 15% drinking more than recommended both at baseline and at 5 years. A baseline comparison with the participants who did not have paired suPAR measurements available (*n* = 3473) showed a higher proportion of females (53.0% vs. 49.4%, *P* = 0.003), younger age (45.6 years vs. 46.4 years, *P* <  0.001), and more daily smokers (43.3% vs. 28.6%, *P* < 0.001) among non-participants.

**Table 1:** Baseline- and 5-year characteristics

	Baseline		5-year visit	
	Mean or N	(SD) or %	Mean or N	(SD) or %
Demographics				
Age (years)	46.4	(7.8)	51.8	(7.8)
Male sex, no.	1632	50.6	–	–
Risk factors				
BMI (kg/m^2^)	26.0	(4.3)	26.4	(4.4)
Total cholesterol (mmol/l)	5.47	(1.0)	5.48	(1.0)
LDL cholesterol (mmol/l)	3.45	(0.9)	3.34	(0.9)
HDL cholesterol (mmol/l)	1.45	(0.4)	1.57	(0.4)
Triglyceride (mmol/l)	1.28	(1.1)	1.31	(1.0)
Systolic blood pressure (mmHg)	130.0	(16.7)	128.8	(16.4)
Diastolic blood pressure (mmHg)	82.5	(11.3)	80.9	(10.3)
Lifestyle				
Diet, no.	3133		3165	
Healthy	481	15.4	834	26.4
Average	2200	70.2	2053	64.9
Unhealthy	452	14.4	278	8.8
Smoking, no.	3201		3210	
Never	1333	41.6	1333	41.5
Former	828	25.9	784	24.4
Occasional	125	3.9	113	3.5
Quit	–	–	271	8.4
Daily	915	28.6	709	22.1
Alcohol intake, no.	3114		3069	
Abstinent	269	8.6	257	8.4
Within recommendations	2379	76.4	2380	77.5
Overuse	466	15.0	432	14.1
Physical activity, no.	3172		3193	
Low activity	635	20.0	485	15.2
Light activity	1971	62.1	2047	64.1
Moderate activity	528	16.6	626	19.6
High activity	38	1.2	35	1.1

Baseline and 5-year characteristics of the main study sample with paired suPAR measurements (*n* = 3225). Abbreviations: *SD* standard deviation

### suPAR changes from baseline to the 5-year visit

The median baseline suPAR (suPAR_0_) was 3.08 ng/ml (IQR 1.29) for men and 3.44 ng/ml (IQR 1.46) for women, and the median 5-year suPAR (suPAR_5_) was 3.29 ng/ml (IQR 1.36) for men and 3.66 ng/ml (*P* < 0.0001, IQR 1.35) for women. suPAR increased significantly over the 5 year period with a similar increase in both men (6.6%, IQR 42.0, P < 0.0001) and women (6.0%, IQR 41.7, P < 0.0001).

### Diet

**Fig. 2:**
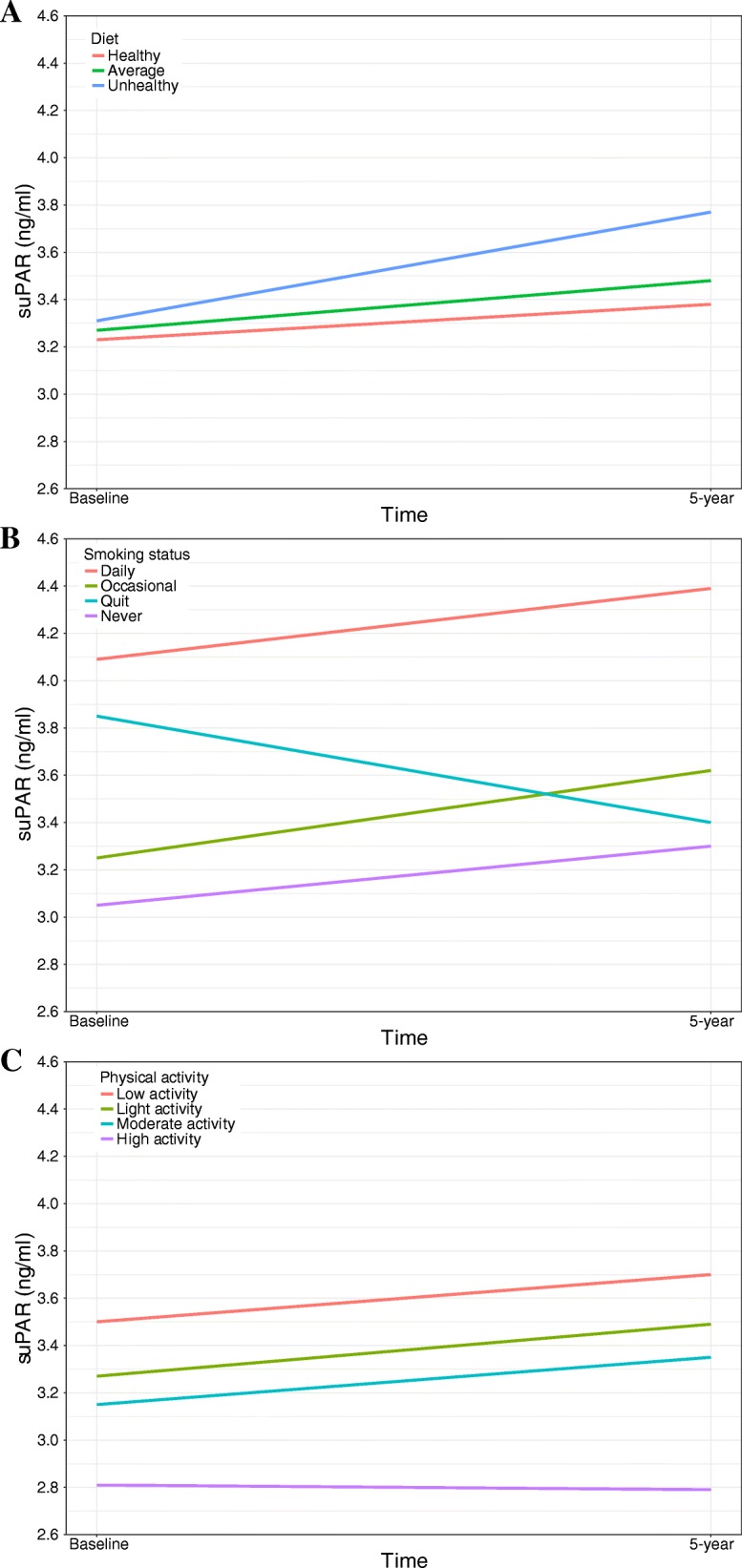
Median suPAR at baseline and at the 5-year visit stratified according to diet (**a**), smoking (**b**), and physical activity (**c**)

In univariate analyses, the median suPAR increase was significantly higher for those with an unhealthy 5-year diet compared with a healthy diet (Table 2, Fig. 2a). To isolate the effect of lifestyle habits on suPAR changes by taking into account confounding, models with appropriate adjustments were constructed. Briefly, suPAR_5_ was modelled as a function of age, sex, suPAR_0_, and lifestyle by multiple linear regression. The back-transformed estimates are interpreted as the percent suPAR change associated with each variable (Table 3). In Model 1, one life style variable was included at a time, whereas in Model 2, all four lifestyle variables were mutually adjusted (see Methods for details on the modelling strategy). When adjusted for suPAR_0_, age, and sex (Table 3, Model 1), an unhealthy diet was associated with significantly higher suPAR_5_ when compared with a healthy diet. With additional adjustments for the intervention group and the other lifestyle factors, the effect was attenuated, but still significant (Table 3, Model 2).

**Table 2:** Median suPAR change from baseline to the 5-year visit stratified according to 5-year lifestyle

	N	suPAR_0_ (IQR)	suPAR_5_ (IQR)	% increase (IQR)	*P*-value
All participants	3225	3.26 (1.41)	3.48 (1.39)	6.3 (41.8)	
Diet rating					
Unhealthy	278	3.31 (1.70)	3.77 (1.86)	10.8 (44.6)	
Average	2053	3.27 (1.43)	3.48 (1.41)	6.2 (41.1)	
Healthy	834	3.23 (1.29)	3.38 (1.26)	3.9 (43.3)	*0.11*
Smoking					
Daily					
➔Daily	642	4.09 (1.83)	4.39 (2.07)	7.0 (40.5)	
➔Occasional	33	3.25 (1.47)	3.62 (1.77)	−7.8 (42.9)	
➔Quit	238	3.85 (2.05)	3.40 (1.31)	−9.1 (46.5)	
Never/occasional/former					
➔Daily	64	3.15 (1.37)	3.55 (1.74)	16.8 (43.8)	
➔Occasional	79	3.31 (1.07)	3.24 (0.96)	1.0 (40.2)	
➔Former	781	3.09 (1.06)	3.30 (1.15)	5.5 (42.5)	
➔Never	1317	3.05 (1.15)	3.30 (1.14)	8.1 (40.9)	
➔Quit	33	3.07 (0.93)	3.47 (1.03)	13.0 (40.4)	*< 0.001*
Alcohol intake, men					
Abstinent	92	3.32 (1.79)	3.30 (1.24)	−0.6 (48.6)	
Within recommendations	1207	3.05 (1.23)	3.26 (1.33)	7.2 (41.2)	
Overuse	285	3.14 (1.36)	3.38 (1.39)	8.1 (43.3)	*0.063*
Alcohol intake, women					
Abstinent	165	3.50 (1.74)	3.89 (1.65)	10.8 (49.6)	
Within recommendations	1173	3.42 (1.44)	3.60 (1.29)	5.3 (41.1)	
Overuse	147	3.41 (1.47)	3.62 (1.49)	5.9 (42.9)	*0.22*
Physical activity					
Low activity	485	3.50 (1.60)	3.70 (1.66)	8.5 (42.4)	
Light activity	2047	3.27 (1.40)	3.49 (1.39)	6.1 (42.2)	
Moderate activity	626	3.15 (1.24)	3.35 (1.25)	5.0 (41.0)	
High activity	35	2.81 (0.78)	2.79 (0.59)	3.5 (49.4)	*0.47*

Median suPAR_0_, suPAR_5_, and % suPAR change from baseline to the 5-year visit stratified according to 5-year lifestyle. ➔: indicates the interaction between baseline- and 5-year smoking habits. *P*-values from the Kruskal-Wallis test performed on the % suPAR change. Abbreviations: CI: confidence interval; IQR: interquartile range; suPAR_0_: baseline suPAR; suPAR_5_: 5-year suPAR

**Table 3:** Percent impact of 5-year lifestyle on 5-year suPAR

	5-year follow-up			
	Model 1		Model 2	
	% more suPAR_5_	95% CI	% more suPAR_5_	95% CI
Demographics				
Age, men (per 5 years)	2.9^***^	(2.0–3.8)	3.1^***^	(2.3–4.0)
Age, women (per 5 years)	0.2	(−0.6–1.1)	0.7	(−0.09–1.6)
Female sex (vs. male sex)	6.9^***^	(4.8–9.0)	8.0^***^	(5.9–10.2)
Lifestyle				
Diet (vs. healthy)				
Unhealthy	11.3^***^	(7.2–15.5)	5.9^**^	(2.0–10.0)
Average	3.8^**^	(1.5–6.1)	1.9	(−0.3–4.2)
Smoking baseline➔5-years (vs. never➔never)				
Daily				
➔Daily	18.8^***^	(15.7–21.9)	17.6^***^	(14.5–20.8)
➔Occasional	3.1	(−5.8–12.9)	4.3	(−4.8–14.1)
➔Quit	−3.1	(−6.6–0.5)	−3.0	(−6.5–0.7)
Never/former/occasional				
➔Daily	10.2^**^	(3.2–17.7)	8.7^*^	(1.8–16.1)
➔Occasional	−3.5	(−9.1–2.4)	−3.6	(−9.2–2.3)
➔Former	−1.4	(− 3.7–0.9)	−1.3	(−3.5–1.1)
➔Quit	4.6	(−4.4–14.5)	5.9	(−3.2–16.0)
Physical activity (vs. high activity)				
Low activity	17.5^**^	(7.0–29.0)	12.8^**^	(3.1–23.5)
Light activity	11.4^*^	(1.8–22.0)	8.8	(−0.4–18.9)
Moderate activity	9.2	(−0.4–19.7)	7.7	(−1.5–17.8)
Alcohol intake, men (vs. recommended)				
Abstinent	0.7	(− 4.9–6.6)	−1.0	(− 6.4–4.7)
Overuse	4.2^*^	(0.6–7.8)	2.7	(−0.8–6.2)
Alcohol intake, women (vs. recommended)				
Abstinent	7.1^**^	(2.5–11.9)	6.6^*^	(2.1–11.3)
Overuse	1.5	(−3.0–6.3)	−0.9	(− 5.2–3.7)

The effect of lifestyle on suPAR_5_ adjusted for suPAR_0_

Model 1: log_2_(suPAR_5_) ~ age, sex, log_2_(suPAR_0_) + one lifestyle factor per line (*n* = 3069–3225)

Model 2: log_2_(suPAR_5_) ~ age, sex, log_2_(suPAR_0_), intervention intensity, diet, smoking, and physical activity (*n* = 3166, R^2^ = 0.326). For categorical variables, the parenthesis indicates the reference value. Model 2 with alcohol was run separately because of missing observations (*n* = 3044, R^2^ = 0.331)

➔: indicates the interaction between baseline and 5-year smoking habits, e.g. never (baseline)➔never (5-year). The horizontal line indicates the split between Model 2 without (above) and with (below) alcohol habits. *: *P* < 0.05, **: *P* < 0.01, ***: *P* < 0.001. Abbreviations: CI: confidence interval; suPAR_0_: baseline suPAR; suPAR_5_: 5-year suPAR

### Smoking

The 238 participants who quit daily smoking since baseline had a median suPAR *decrease* of 9.1% compared with an 8.1% *increase* among never smokers (Table 2, Fig. 2b). In Model 1, smoking habits were strongly associated with suPAR_5_ levels, with continuous daily smokers having significantly higher suPAR compared with the other categories (Table 3, Model 1). No significant difference was observed between never smokers and occasional smokers (Table 3, Model 1). Interestingly, those who quit daily smoking had suPAR_5_ levels comparable to never smokers (daily➔quit vs. never, *P* = 0.11). The additional adjustments in Model 2 did not affect the estimates (Table 3, Model 2).

### Alcohol

Alcohol consumption was not significantly associated with suPAR changes in univariate analyses (Table 2). In Model 1, alcohol overuse in men was associated with higher suPAR_5_ when compared with a moderate intake (*P* = 0.022). In women, abstinence was associated with higher suPAR_5_, with no difference between overuse and moderate intake (Table 3, Model 1). In the fully adjusted Model 2, only the association for women remained.

### Physical activity

The univariate analysis showed a non-significant trend towards less of an increase in suPAR with higher physical activity (Table 2, Fig. 2c). In adjusted analyses, low physical activity was significantly associated with higher suPAR_5_ levels (Table 3, Model 1). In Model 2, the effect was attenuated, but still significant (Table 3, Model 2).

### Model control

**Fig. 3:**
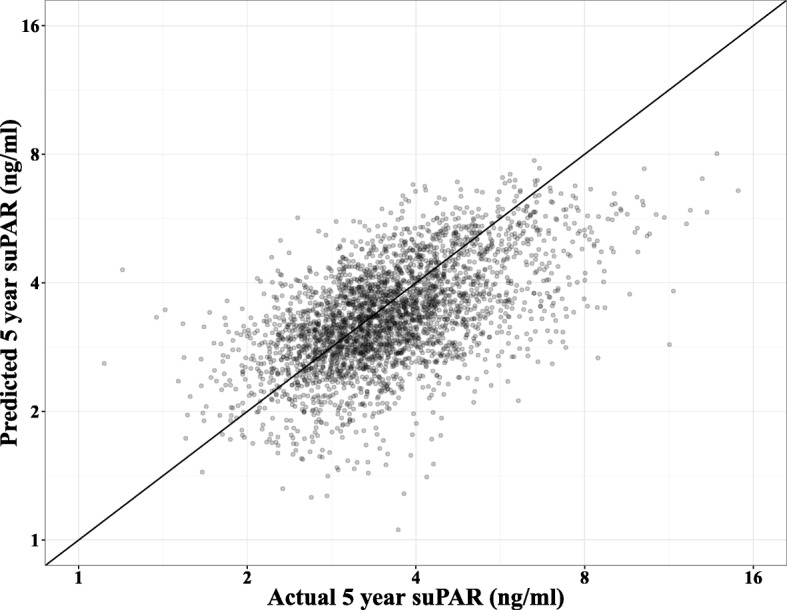
Predicted 5-year suPAR values from Model 2 (log_2_(suPAR_5-year_) ~ log_2_(suPAR_baseline_), sex, age, intervention group, diet, smoking, physical activity) plotted against the actual 5-year suPAR values. Note the logarithmic axes. Thin line: indicates agreement

To visualize the quality of the multiple linear regression models, predicted suPAR_5_ values were generated using the final Model 2 (without alcohol). Plotting the model predictions against the actual suPAR_5_ values (Fig. 3) showed fair agreement between the actual and predicted suPAR_5_. However, the model systematically over-estimated low suPAR_5_ values and under-estimated high values.

### 5-year suPAR level and mortality

**Fig. 4:**
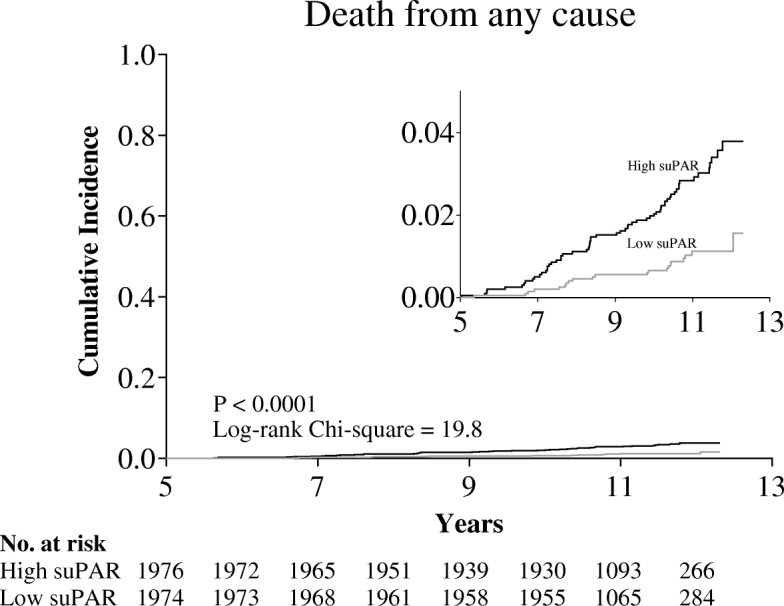
Mortality after the 5-year visit stratified according to age- and sex-specific 5-year suPAR median splits. The insert indicates a zoom to the 0.00 to 0.04 mortality range. Low suPAR range: 0.90–3.76 ng/ml. High suPAR range: 3.09–15.4 ng/ml

All 3950 participants with a suPAR_5_ measurement and complete smoking history were followed for a mean of 6.1 years after the 5-year visit, during which 82 subjects (2.08%) died. When stratifying the participants according to age- and sex-specific suPAR_5_ median splits, the group with high suPAR_5_ (mean 4.73 ng/ml, range 3.09–15.4 ng/ml) had a mortality of 3.8% compared with 1.6% in the low suPAR_5_ group (mean 2.93 ng/ml, range 0.90–3.76 ng/ml), *P* < 0.001 (Fig. 4). In Cox regression analyses, the hazard ratio (HR) per suPAR_5_ doubling was 2.42 (95% CI: 1.58–3.72) when adjusted for age and sex. Even with additional adjustments for all four lifestyle factors, the HR for mortality for a suPAR_5_ doubling remained significant (Table 4). Only 5-year dietary habits had a significant impact on mortality (HR 0.37 for a healthy diet vs. an unhealthy diet, *P* = 0.04) in the fully adjusted model; whereas, the effect of 5-year smoking habits was non-significant when suPAR was included in the model (data not shown).

**Table 4:** Hazard ratios for mortality per suPAR_5_ doubling

Adjustments	N (deaths)	HR per suPAR_5_ doubling (95% CI)
None	3950 (82)	2.48 (1.64–3.76)
Sex, age	3950 (82)	2.42 (1.58–3.72)
Sex, age, smoking	3934 (82)	2.17 (1.35–3.48)
Sex, age, smoking, diet, exercise	3905 (80)	2.19 (1.35–3.54)
Sex, age, smoking, diet, exercise, alcohol	3734 (74)	2.03 (1.22–3.37)

Hazard ratio (95% CI) for all-cause mortality per doubling of suPAR_5_ with step-wise adjustments for sex, age, and 5-year lifestyles. Abbreviations: CI: confidence interval; suPAR_5_: 5-year suPAR

## Discussion

In this large study of individuals from the general population measured at baseline and after 5 years—and followed for 6 years hereafter—we demonstrated that major lifestyle habits (diet, smoking, alcohol, and physical activity) are reflected in the inflammatory biomarker suPAR, with healthy habits generally associated with lower suPAR and unhealthy habits with higher suPAR. The absolute suPAR_5_ level, in turn, was strongly associated with mortality, even when adjusted for the lifestyle habits. From baseline to the 5-year visit, suPAR increased by a median of 6%. Unhealthy lifestyle habits were generally associated with a larger suPAR increase compared to healthy lifestyle habits in univariate analyses.

Studies in both the general population and patient studies have shown that suPAR is a strong marker for mortality. The current study suggests that suPAR is a modifiable risk factor, an early warning signal and not a death sentence. But what does the suPAR level reflect? suPAR shows a positive correlation with CRP [21]. However, suPAR and CRP seem to reflect different aspects of inflammation, and it has been proposed that suPAR reflects cellular inflammation while CRP reflects metabolic inflammation [26]. While CRP is rapidly up- and down-regulated, suPAR show stable kinetics with limited circadian fluctuation [18, 27] and, as shown in the current paper, with a high correlation over a 5-year period. Furthermore, suPAR is positively correlated with pro-inflammatory cytokines, including IL-1β, IL-6, and TNF-α [28], all of which are upregulated during the process of inflammaging [29]. On a transcriptional level, activation of pattern recognition receptors, i.e., Toll-like-receptors and NOD-like receptors (e.g. by smoking [30]), as well as proinflammatory cytokine receptors activate the transcription factors NF-κB and AP-1 that bind the promoter region of the gene for uPAR/suPAR, *PLAUR*, and upregulate expression of uPAR [31]. In contrast, stimulation of whole blood with suPAR has little or no effect on the expression of inflammatory cytokines [32]. Hence, suPAR may be a less functionally active biomarker and therefore allowed to freely circulate, in contrast to most proinflammatory cytokines with strong local acting immune effects.

It could be speculated that suPAR, due to its stability and upregulation during states of even slightly increased immune activity, is a marker of the overall immune activation and inflammatory processes in an individual and therefore a marker of chronic inflammation. While cleavage of uPAR to suPAR may simply be a way to downregulate uPAR activity, suPAR has been suggested to play a role in chemotaxis [33], neutrophil efferocytosis [34], and angiogenesis [35], although the direct immunological effects of suPAR are less well understood.

Whether suPAR is reflective of disease processes or is causing disease is an ongoing discussion [36]. Recent work has shown that suPAR is causal of kidney disease [37], but this observation does not explain why suPAR also predict development of a range of other diseases. In any case, whether suPAR is causal or reflects a disease process, our current study corroborates elevated suPAR as a marker of negative outcome but add the effect of lifestyle and lifestyle interventions on suPAR levels.

To show the actual effect of the four lifestyle factors on suPAR_5_ levels, we modeled the suPAR_5_ values adjusted for suPAR_0_, demographic factors, and the four lifestyle factors to neutralize the regression towards the mean. Interestingly, baseline diet, physical activity, and alcohol consumption had no impact on suPAR_5_, probably because the baseline habits are adequately reflected in suPAR_0_. In contrast, a smoking history was necessary for the model, because smoking is likely to be the lifestyle factor with the highest impact on suPAR level. Despite the high impact of daily smoking on suPAR levels, we found no difference in suPAR level change between never smokers and occasional smokers. We recently found that a high degree of self-control is associated with lower suPAR levels [38], which may in part explain why occasional smoking is not associated with higher suPAR levels. The fully adjusted model showed that when comparing men identical at baseline, with the same suPAR_0_, but with very different 5-year lifestyles, the combination of unhealthy habits within all four lifestyle areas at 5 years was associated with 44% higher suPAR_5_ (1.059 × 1.176 × 1.027 × 1.128 = 1.44, estimates from Table 3) when compared with healthy habits. The model predictions (Fig. 3) indicate that this difference is likely to be underestimated, probably because the assumption of an independent effect of each lifestyle factor is not realistic; instead, there may be a synergistic effect of clustering several unhealthy or healthy habits.

When following the participants for approximately 6 years after the 5-year visit, the absolute suPAR_5_ level was strongly associated with all-cause mortality. When adjusted for suPAR_5_, smoking habits, alcohol consumption, and physical activity had no effect on mortality. Taken together, suPAR was a strong predictor of mortality in this cohort, but the lifestyle habits that affect suPAR were not. This finding is highly interesting, as it suggests that a given lifestyle modification’s impact on mortality risk can be assessed by performing a subsequent suPAR measurement. Further studies are needed to confirm that serial suPAR measurements can be used as a guide for the “true” risk reduction of lifestyle changes.

This study has several strengths. The paired nature of the suPAR measurements strongly supports the causality between certain lifestyle habits and suPAR, since any lifestyle changes always occurred before the second suPAR measurement. The modeling framework ensured that the effects of the lifestyle habits were mutually adjusted, and regression towards the mean was eliminated by adjusting for baseline suPAR. Also, the effects of lifestyle found in this study are generally in agreement with the baseline study results [25]. The recording of mortality is highly accurate as all deaths of persons living in Denmark are recorded in national registries. All lifestyle habits were recorded via validated questionnaires.

A limitation of the study is that less than half of the participants had available suPAR measurements both at baseline and the 5-year visit, and the proportion of daily smokers among participants was considerably lower. Thus, it is likely that more resourceful and relatively healthy persons participated in the 5-year visit. For self-reported lifestyle habits, we were only able to exclude those misreported for the obviously impossible combinations of smoking habits (e.g. a daily smoker at baseline cannot be a never smoker after 5 years). We suspect that social desirability bias led to a systematic under-reporting of unhealthy habits. It is also likely that certain combinations of lifestyle habits affect suPAR in a synergistic way, as suggested by the systematical over- and under-estimation of high and low suPAR levels, respectively.

## Conclusion

We demonstrated that suPAR covaries with lifestyle in a large general population cohort, with healthy 5-year lifestyle habits associated with lower suPAR levels. The models indicate that serial suPAR measurements can be interpreted using just one previous measurement, information about current lifestyle, and smoking history. For the almost four thousand subjects with suPAR_5_ values, suPAR was highly predictive of mortality during the next 6 years, irrespective of their lifestyle. We propose that suPAR is a candidate biomarker not only for lifestyle changes, but also for the subsequent risk of mortality.

## Methods

### Charaterization of participants

The current study population consisted of participants in a large population-based study, Inter99, which is reported in detail elsewhere [25, 39, 40]. Briefly, 61,301 individuals from a general population, aged 30 to 60 years and living in the southwestern part of Copenhagen were randomly selected to participate in the study. There were 13,016 subjects randomized to the intervention group and 6784 (52.5%) attended the clinic at baseline. All participants received repeated individual lifestyle counseling on smoking habits, physical activity, alcohol consumption, and dietary habits, and the majority (90%; high-intensity group A) were also offered repeated group-based lifestyle counseling. Participants in the low-intensity group B (10%) only received repeated individual lifestyle counseling.

From 1999 to 2000, 5538 participants (81.6%) had suPAR measured at baseline (suPAR_0_) [25]. Five years later in 2004–2005, they were invited to the 5-year visit: 3678 participated (66.4%), and 3311 (90.0%) had their suPAR re-measured (suPAR_5_, Fig. 1). Eighty-five had an invalid smoking history (e.g. never smoking at 5 years, but daily smoking at baseline) and were excluded from further analyses. The group of baseline never smokers who reported daily smoking at 5 years only included one participant who was also excluded. Thus, the study main sample comprised 3225 participants. An additional 725 participants had suPAR_5_ measurements, but no suPAR_0_ measurement, and were included in the suPAR_5_ survival analyses.

### Assessments

The serum obtained in 1999–2000 (baseline) and in 2004–2005 (5-year) were collected the following way: Three 7 ml Becton Dickinson tubes (no. 367789) were filled with blood and centrifugated for 25 min at 1300G. Serum was transferred to a Sarstedt tube (no. 60540012) and stored at − 20 °C until measurement of suPAR. The serum suPAR level was measured with the suPARnostic ELISA (ViroGates A/S, Birkerød, Denmark) in 2011. Participants with serum suPAR higher than 22 ng/ml (outside assay range) or an increase from baseline to the 5-year visit higher than 20 ng/ml were excluded (*n* = 5). All participants answered a questionnaire on lifestyle and underwent a physical examination at baseline and at the 5-year visit. The methods used for measuring body mass index (BMI), cholesterol, triglycerides, and blood pressure were described by Jørgensen and coworkers [39]. Lifestyle was self-reported. The dietary quality score (DQS-9) is validated and based on a one-week food diary where four food groups (vegetables, fruit, fish, and fat) were given points based on the degree of compliance with national dietary recommendations, and the combined score was categorized as “unhealthy”, “average”, or “healthy” diet [41]. Self-reported smoking habits were registered (“never”, “former”, “occasional”, or “daily” smokers) and an extra category was created for those who quit smoking at the 5-year visit. Weekly self-reported consumption of alcoholic beverages was converted into units (12 g) of alcohol per week and categorized according to national recommendations in 1999 as “abstinent”, “within recommendations” (≤ 14 units/week for women and ≤ 21 units/week for men) or “overuse” (> 14 units/week for women and > 21 units/week for men). Leisure time physical activity was reported according to the categories by Saltin: mainly reading or watching television or equivalent sedentary activities (“low”); going for walks, biking, or equivalent light activity for up to 4 h per week (“light”); sport activities at least three times per week or an equivalent amount of strenuous gardening or similar (“moderate”); or competitive sports or long distance running several times per week (“high”) [42].

### Statistical analysis

The suPAR change from baseline to the 5-year visit was calculated as the ratio of suPAR_5_/suPAR_0_ and listed in Table 2 as the median percent suPAR change. Differences in suPAR ratios were tested with the Kruskal-Wallis test. For the adjusted models, log_2_-transformed suPAR_5_ was modeled by multiple linear regressions as a function of log_2_-transformed suPAR_0_, demographic variables, and lifestyle variables. The estimates were back-transformed by (2^β^–1) × 100 and interpreted as the percent difference in suPAR_5_. All models were adjusted for suPAR_0_, sex, and age at the 5-year visit, and they included the interaction between age and sex observed in the baseline study [25]. The models with alcohol consumption as an explanatory variable were analyzed separately because of more missing observations for alcohol (*n* = 156), and we allowed for the interaction between sex and alcohol consumption observed in the baseline study. Models with smoking included an interaction between the 5-year smoking habits and dichotomized baseline smoking habits (daily smoker or not daily smoker). Tables 2 and 3 are stratified in accordance with these interactions.

The survival analyses are Cox proportional hazards models with death as the event, years after the 5-year visit as the time variable, and log_2_(suPAR_5_) as the main explanatory variable. For the Kaplan Meier plot, we generated sex- and age-specific (≤ 55 years or > 55 years) suPAR_5_ median splits. All tests were two-sided and interpreted at an α-level of 0.05. SAS 9.4 (SAS Institute, Cary, NC, USA) was used for statistical analysis.

### Modeling strategy

**Fig. 5:**
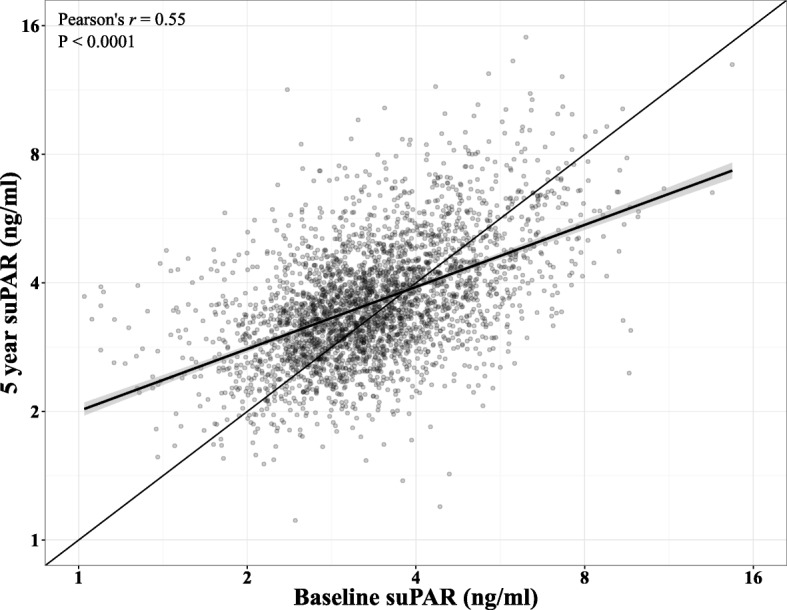
The 5-year suPAR plotted against the baseline suPAR with linear trend line and Pearson’s correlation coefficient. Note the logarithmic axes. Thick line: Loess line with 95% confidence interval. Thin line: indicates agreement

The interpretation of raw suPAR changes was hampered by regression towards the mean, i.e. a high suPAR_0_ was generally associated with a lower suPAR_5_ (Fig. 5). Consequently, all models of suPAR_5_ were adjusted for suPAR_0_. For each lifestyle variable (diet, smoking, alcohol, and physical activity), models were fitted with 5-year and baseline lifestyle interactions and 5-year and baseline lifestyle without interactions. A significant interaction was found for smoking (*P* = 0.014). For all other lifestyle factors, only the 5-year lifestyle had an effect on suPAR_5_. We generated two model frameworks. In Model 1, suPAR_5_ was modeled as a function of age, sex, suPAR_0_, and one lifestyle variable at a time. In Model 2, all four lifestyle factors and the intervention intensity were included in the same model (estimates for intervention intensity not shown as they had no significant effect on suPAR).

## Abbreviations

CRP: C-reactive protein
CVD: Cardiovascular disease
DQS-9: Dietary quality score
suPAR: Soluble urokinase plasminogen activator receptor
suPAR_0_: Baseline suPAR level
suPAR_5_: 5-year suPAR level
uPAR: Urokinase plasminogen activator receptor
